# Intrasubject functional connectivity related to self‐generated thoughts

**DOI:** 10.1002/brb3.1860

**Published:** 2020-12-15

**Authors:** Daniel Brennan, James W. Murrough, Laurel S. Morris

**Affiliations:** ^1^ Department of Psychology Graduate School of Arts & Sciences NYU New York NY USA; ^2^ Depression and Anxiety Center for Discovery and Treatment Department of Psychiatry Icahn School of Medicine of Mount Sinai New York NY USA

**Keywords:** data‐driven analysis, functional connectivity, mind‐wandering, network‐based statistic, repeated measures

## Abstract

**Introduction:**

In psychiatric research, functional connectivity (FC) derived from resting‐state functional MRI (rsfMRI) is often used to investigate brain abnormalities in psychiatric disorders. This approach assumes implicitly that FC can recover reliable maps of the functional architecture of the brain and that these profiles of connectivity reflect trait differences underlying pathology. However, evidence of FC related to self‐generated thoughts (mind‐wandering) stands in contrast with these assumptions, as FC may reflect thought patterns rather than functional architecture.

**Methods:**

Multi‐factor analysis (MFA) was used to investigate the reported content of self‐generated thoughts during high‐field (7T) rsfMRI in a repeated sample of 22 healthy individuals. To investigate the relationship between these experiences and FC, individual scores for each of these dimensions were compared with whole‐brain connectivity using the network‐based statistic (NBS) method.

**Results:**

This analysis revealed three dimensions of thought content: self‐referential thought, negative thoughts about one's surroundings, and thoughts in the form of imagery. A network of connections within the sensorimotor cortices negatively correlated with self‐generated thoughts concerning the self was observed (*p* = .0081, .0486 FDR).

**Conclusion:**

These results suggest a potentially confounding relationship between self‐generated thoughts and FC, and contribute to the body of research concerning the functional representation of mind‐wandering.

## Graphical abstract

In psychiatric research, functional connectivity (FC) derived from resting‐state functional MRI (rsfMRI) is often used to investigate brain abnormalities in psychiatric disorders; this approach assumes implicitly that FC can recover reliable maps of the functional architecture of the brain, and these profiles of connectivity reflect trait differences underlying pathology. However, evidence of FC related to self‐generated thoughts (mind wandering) stands in contrast with these assumptions, as FC may reflect thought patterns rather than functional architecture. Here, multi‐factor analysis (MFA) was used to investigate the reported content of self‐generated thoughts during high‐field (7T) rsfMRI in a repeated sample of 22 healthy individuals: this analysis revealed a network of connections within the sensorimotor cortices negatively correlated with self‐generated thoughts concerning the self (*p* = .0081, .0486 FDR), which suggests a potentially confounding relationship between self‐generated thoughts and FC.

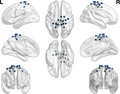

## 1 INTRODUCTION

Resting‐state functional magnetic resonance imaging (rsfMRI) is a common experimental paradigm utilized in both cognitive neuroscience and psychiatric research which measures endogenous fluctuations of the blood‐oxygen level dependent (BOLD) signal under resting conditions. The co‐occurrence of spatially distinct signals over time, called functional connectivity (FC), can be measured using this method and signifies a functional relationship between two or more distinct areas of the brain. In psychiatric research, the presence and magnitude of resting FC in populations of interest are used to investigate dysfunctional brain development and functional disorganization related to pathology (Baldassarre et al., 2016; Bassett & Bullmore, 2009). This approach relies implicitly on the ability of FC to recover reliable maps of the functional architecture of the brain (Gratton et al., 2018), and that these profiles of connectivity reflect trait differences underlying pathology. The interpretation of resting FC in psychiatry therefore fundamentally relies on the relationship between the resting FC signal with underlying brain organization. With regards to pathology, connectivity derived through the resting‐state approach is thought to reflect an “intrinsic functional architecture” which is suitable for comparison between clinical populations of interest and controls (Guerra‐Carrillo et al., 2014; Redcay et al., 2013).

There is evidence that some functional networks derived using rsfMRI can be recovered using electrophysiological methods (Kucyi et al., 2018; Raccah et al., 2018). Networks are also reliably detectable across various conscious states and tasks (Rosazza & Minati, 2011), supporting the hypothesis that resting FC reveals information about the intrinsic organization of the brain. Despite this evidence, the interpretation of FC as “intrinsic” and constrained by meaningful brain organization is highly contested (Honey et al., 2009). FC is an inherently ambiguous measure (Reid et al., 2017, 2019) and can be present without direct anatomical connection which likely reflects a purely functional relationship mediated by an indirect pathway (Damoiseaux & Greicius, 2009).

A further source of variability in the FC signal is evidenced by mind‐wandering research. Empirical evidence of FC related to task state, cognitive state, or self‐generated thoughts (mind‐wandering) demonstrates that FC is a malleable signal which can be modulated by psychological phenomena and behavior. FC is therefore susceptible to the influence of “task” demands (Yao et al., 2012), and these modulations may vary across individuals (Gratton et al., 2018), creating a task‐subject interaction on the ultimate measurement of FC. It is therefore possible that FC may be *systematically* influenced across pathologies by the content of self‐generated thoughts; in populations where altered thought content is central to the disorder, it follows that FC may reflect these thought patterns, not intrinsic functional architecture necessarily (Damoiseaux & Greicius, 2009).

The nature of these resting‐state networks is integral as to the interpretability of functional connectivity in psychiatry. If they are truly indicative of functional organization, they should be stable across observations and robust to passing thoughts while measurement occurs. Conversely, if changes to FC are observable within individuals as a function of thought content, this would stand as evidence that FC is not fully reliable as a measure of functional architecture and reveal a possible confound for rsfMRI studies which include groups with categorically different patterns of thought. Therefore, it is imperative to investigate the variance of self‐generated thoughts both across and within individuals, and the relationship of these thoughts with FC.

## 2 MATERIALS AND METHODS

Ultrahigh field resting‐state fMRI, T1 structural images, and a postscan questionnaire regarding self‐generated thoughts are made publicly available by the Max Planck Institute (Gorgolewski et al., 2015); scan parameters are reproduced below. Twenty‐two (22) neurotypical, native German‐speaking participants were scanned on two separate occasions, spaced 1 week apart. Each session included two 1.5 mm isotropic functional scans covering the whole brain and one submillimeter scan of the prefrontal cortex (not utilized in this analysis). Structural T1 images and Field Map Images, for estimating B0 inhomogeneities, were also acquired. Physiological and phenotypic data included: measures of mood, sustained attention, blood pressure, respiration, pulse, and the content of self‐generated thoughts via a shortened version of the New York Cognitive Questionnaire (NYCQ, see Table S1; (Gorgolewski et al., 2014). The final dataset consisted of the 4 full‐brain fMRI scans of 19 different individuals (10 women); only participants with all 4 usable scans were included in the analysis due to the permutation methods of the repeated measures GLM in the fMRI analysis.

### 2.1 Behavioral statistical analysis

To investigate the content and stability of self‐generated thoughts in this sample, multiple factor analysis (MFA), a variant of principal components analysis (PCA), was used analyze the variance structure of the behavioral responses. Like PCA, MFA aims to calculate new variables, called *principal components*, which exist as linear combinations of the original variables (Abdi & Williams, 2010). These new variables are interpreted as the thought content which was experienced within the scanner across this sample.

These new variables are best described by their *factor loadings. Factor loadings* describe how the original variables “load” onto these new factors, and thus represent which of the variables from the original dataset make up this new *component*. Each retained *component* therefore represents combinations of original variables which covary together and likely describe a unique dimension of experience within the scanner. Each observation also has a *factor score* for each new component, which provides information regarding which observations are most associated with the individual factors, or the degree to which the component is represented at that observation. Both the *factor scores* and *factor loadings* of included *components* can be used to provide a consolidated description of the original dataset, with the added bonus of values for each *component* at each observation from which further analysis may be conducted.

Multi‐factor analysis provides an extension to PCA which allows for the analysis of repeated‐measures data structured in a multi‐block format (Abdi et al., 2013). The assumption of independent observations for traditional PCA is not met for this study due to the repeated measures facet of the dataset. To account for this, the observations at each time point were first analyzed independently as separate data “blocks” so that they may be normalized by their first *eigenvalue* and contribute equally, in terms of their variance, to the grand data structure. Furthermore, the data were normalized via a “nested” method: In this specific dataset, the inertia of each “day” (block 1&2, block 3&4) and “time‐point” (block 1&3, block 2&4) is also added as normalization factors. After this normalization process, the analysis procedure and output are analogous to standard PCA and can be interpreted accordingly.

Interpretation *of factor loadings* is a controversial point in PCA analysis. It is not always clear how many of the original variables to include as *factor loadings* for the new calculated component, and therefore it is quite easy to over‐interpret the distribution of loadings. A benefit of multi‐block MFA is the ability to assess stability of factor loadings across “blocks” or, in this case, time points (Abdi et al., 2013). A bootstrapping approach was utilized to reliably assess the content of self‐generated thoughts in this sample by generating confidence intervals for each *factor loading*. *Factor loadings* for the 12 NYCQ variables across all 4‐time points were transformed into *factor contributions* by normalizing the squared *factor loading* by the associated *eigenvalue*, revealing the proportion of variance explained by each factor at each time point. These *contributions* therefore sum to 1, and the additive property of these *contributions* was used to create confidence intervals from which the reliability of these loadings may be assessed (Abdi et al., 2013).

The null distribution of bootstrapped *contributions* for each of the original 12 items of the questionnaire was used to assess the strength and stability of these loadings. Because these contributions are additive, the expected value of any given contribution summed over the 4‐time points is 1/12 = 0.083. Confidence intervals were calculated by observing the values which bounded 95% of the null distribution. *Contributions* with a confidence interval that does not include the expected value of 0.083 were considered to reliably contribute to the given component. In this way, a factor loading must be consistently influential across four‐time points to be considered a reliable contributor to that component.

### 2.2 MRI acquisition

Image acquisition parameters reproduced from the data source, Gorgolewski et al., (2015): To obtain T1 images, a 3D MP2RAGE29 sequence was used: 3D‐acquisition with field of view 224 × 224 × 168 mm^3^ (H‐F; A‐P; R‐L), imaging matrix 320 × 320 × 240, 0.7  mm^3^ isotropic voxel size, Time of Repetition (TR) = 5.0 s, Time of Echo (TE) = 2.45 ms, Time of Inversion (TI) 1/2 = 0.9 s/2.75 s, Flip Angle (FA) 1/2 = 5°/3°, Bandwidth (BW) = 250 Hz/Px, Partial Fourier 6/8, and GRAPPA acceleration with iPAT factor of 2 (24 reference lines). Functional scans were obtained with using a 2D sequence: axial orientation, field of view 192 × 192 mm^2^ (R‐L; A‐P), imaging matrix 128 × 128, 70 slices with 1.5 mm thickness, 1.5 mm^3^ isotropic voxel size, TR = 3.0 s, TE = 17 ms, FA = 70°, BW = 1,116 Hz/Px, Partial Fourier 6/8, GRAPPA acceleration with iPAT factor of 3 (36 reference lines), and 300 repetitions resulting in 15 min of scanning time. For estimating B0 inhomogeneities, a 2D gradient echo sequence was used. It was acquired in axial orientation with field of view 192 × 192 mm^2^ (R‐L; A‐P), imaging matrix 64 × 64, 35 slices with 3.0 mm thickness, 3.0 mm^3^ isotropic voxel size, TR = 1.5 s, TE1/2 = 6.00 ms/7.02 ms (which gives delta TE = 1.02 ms), FA = 72°, and BW = 256 Hz/Px.

### 2.3 MRI preprocessing

The skull of anatomical T1 images was removed using an automatic skull stripping process available through AFNI (Cox, 1996). To improve performance of automatic skull stripping, a lower resolution image was intensity‐normalized and stripped using this automated process. The results of this procedure were masked and applied to the higher resolution/contrast image.

Standard signal correction was applied via AFNI’s preprocessing wrapper, afni_proc.py (Cox, 1996), which performs despiking, physiological regression, t‐shifting (aligning a single 3d image in time), spatial alignment to a standard template (Montreal Neurological Institute, MNI), spatial blurring (6 mm), bandpass filtering (0.01–0.1 hz), and motion correction. Spatial distortion correction of EPI images due to B0 inhomogeneities was calculated and applied with FSL’s FUGUE (Jenkinson, Beckmann, Behrens, Woolrich, & Smith, 2012). Physiological regression of heart rate and respiration was calculated with RETROICOR (Glover et al., 2000) and applied through the preprocessing wrapper.

An alignment procedure was applied to the resting‐state data which seeks to minimize total spatial transformations and reduce interpolation errors (Mahmoudzadeh & Kashou, 2013). Alignments were calculated in reverse (anatomical to EPI), inversed, and combined with other alignments (anatomical to shared space) to result in a single transformation which brings the final EPI time course into alignment with the standard spatial template. Furthermore, this transformation has been combined with motion correction within each subject, so that each TR of the time course is aligned to the minimum outlier; this alignment is combined with the overall alignment of the EPI to standard space, resulting in minimum total rotations and therefore minimum interpolation error.

### 2.4 Data‐driven connectivity analysis

To create the connectivity matrix for each participant at each time point, whole‐brain time series data were spatially binned into 470 functional gray‐matter regions, as defined by the Harvard‐Oxford atlas parcellated into 470 regions (Baek et al., 2017; Patel & Bullmore, 2016). These 470 time series were correlated pairwise, so that a connectivity value (Pearson R) exists for each potential connection across the entire brain.

A data‐driven approach was utilized to determine significant functional networks based on the connectivity matrix, related to the content of self‐generated thoughts via the network‐based statistics method (NBS; Zalesky et al., 2010). As opposed to a more traditional seed‐based connectivity analysis, NBS does not require an a‐priori region as the seed from which whole‐brain correlations are calculated. Instead, NBS utilizes a whole‐brain connectivity matrix to create representations of connectivity for each fMRI scan.

Network‐based statistic utilizes a permutation‐based general linear model (GLM) for significance testing (Freedman & Lane, 1983). This method creates a null distribution of networks, the sizes of which are recorded. The empirically observed graphical networks’ sizes are then tested against this null distribution for significance; networks significantly larger than chance are retained. Nuisance predictors are first regressed out against these networks, and the residuals of this regression are permuted against other networks to create the null distribution of networks which consist of connections that survive a primary threshold. This method of permutation is beneficial because some of the variance of the networks across observations might be explained by the nuisance predictors (Anderson & Robinson, 2001). Furthermore, this method allows for more elegant control of family‐wise error rate, a common problem in mass‐univariate testing of whole‐brain signals (Zalesky et al., 2010).

*Factor scores*, calculated via MFA in the behavioral analysis, serve as predictors in the GLM. MFA reveals two sets of *factor scores*: a “*compromise factor score*,” a *factor score* for each individual, and a “*partial factor score*,” a *factor score* for each individual at each time point. The compromise factor scores are the barycenter of all of the partial factor scores (Abdi et al., 2013), and thus are the mean of all the factor scores for each table. These different factor scores can be used to compute between‐subject and within‐subject changes in connectivity. The partial factor scores were therefore used in this analysis, and each individual subject's FC was added as nuisance predictors in the design matrix. Permutation was constrained within each subject's four scans to reveal intra‐subject changes in FC related to that individual's changing thought content.

## 3 RESULTS

### 3.1 Behavioral analysis

**FIGURE 1:**
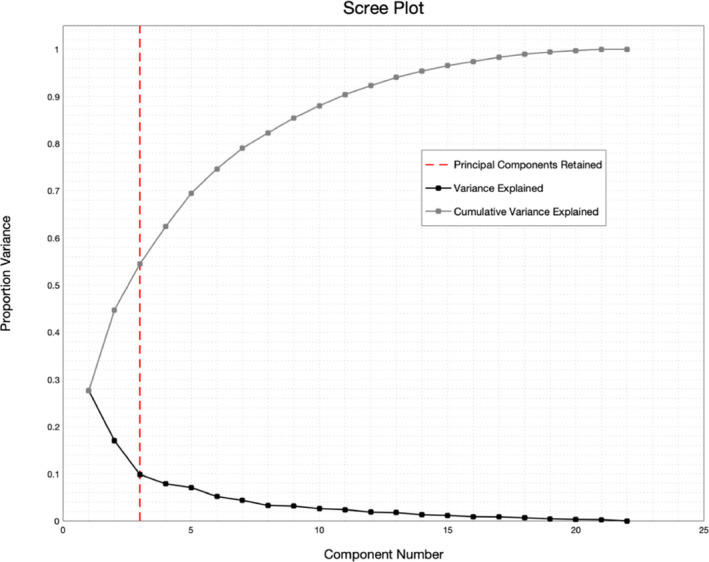
Dashed line indicates point of “elbow” at the last retained principal component. cumulative variance explained by these three components = 54.5%

**FIGURE 2:**
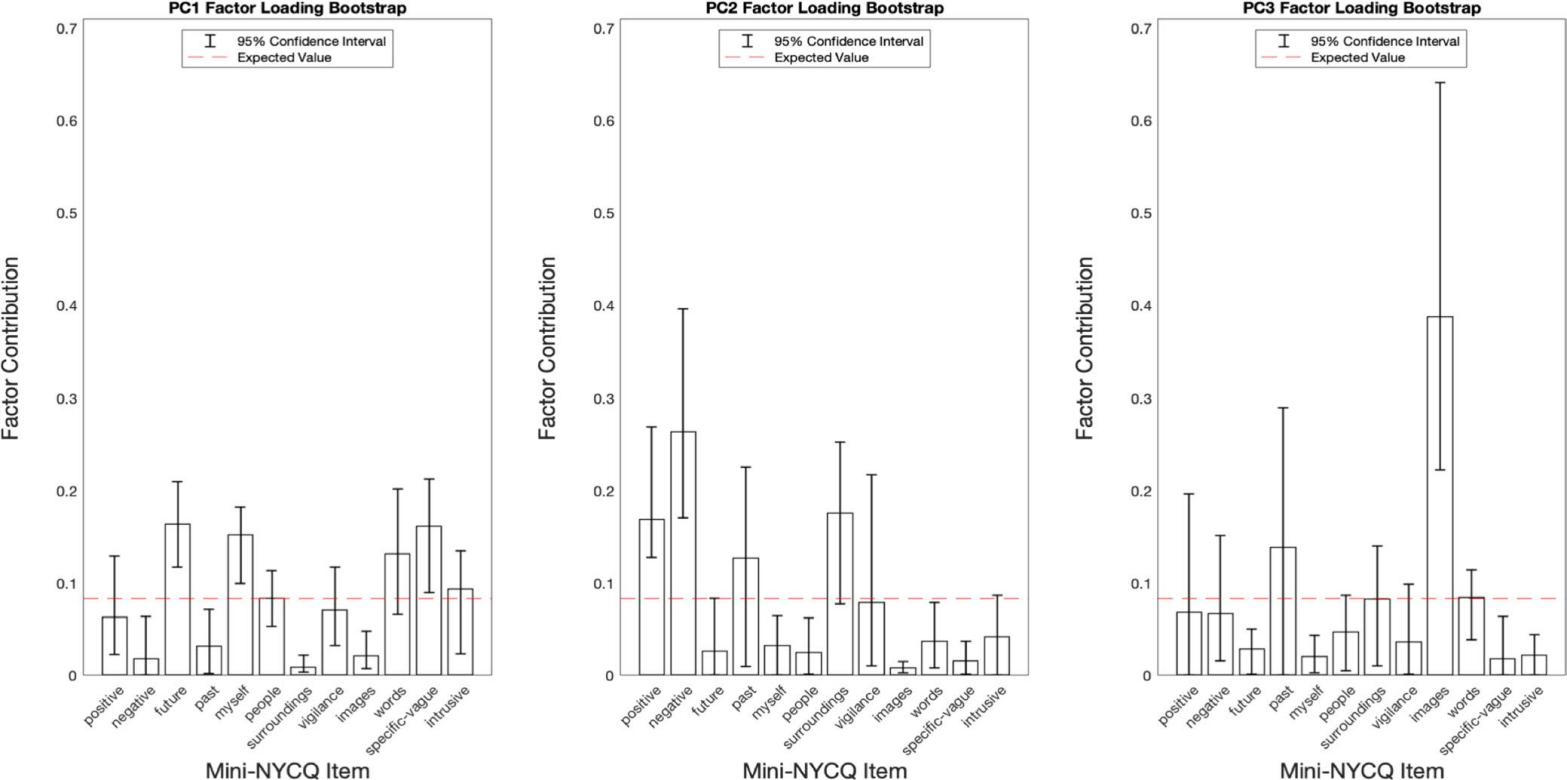
95% confidence interval (Bonferonni Corrected), determined using a bootstrap approach, resampling with replacement factor loadings for each time point to create a confidence interval for the mean factor loading. Factor loadings with CI which did not include the expected value of equal contribution, 8.3%, are considered significant loadings for that given factor

Examination of the scree plot (Figure 1.) reveals an elbow at the 3rd *principal comp*onent. The first 3 *components* explain a total of 54.5% of the variance of the entire behavioral dataset. The *factor loadings* and *contributions* of these three *factors* were therefore investigated, as well as the corresponding *factor scores* were used as predictors in the whole‐brain connectivity analysis. Bootstrap analysis of *factor loadings* revealed significant loadings (Lower Bound Bootstrap Confidence Interval > 8.3% variance explained per component; Figure 2) for the first (PC1), second (PC2), and third (PC3) components. Loadings for the first component were thoughts surrounding the self (Lower Bound = 9.96%), future (Lower Bound = 11.68%) and were specific (Lower Bound = 8.94%). Loadings for the second component were thoughts carrying negative valence (Lower Bound = 17.02%); while not significant after Bonferroni correction, thoughts concerning an individual's surroundings were also strongly associated with this factor (Lower bound = 7.76%). Loadings for the third component were thoughts in the form of images (Lower Bound = 22.23%).

### 3.2 Functional representations of mind‐wandering

**FIGURE 3:**
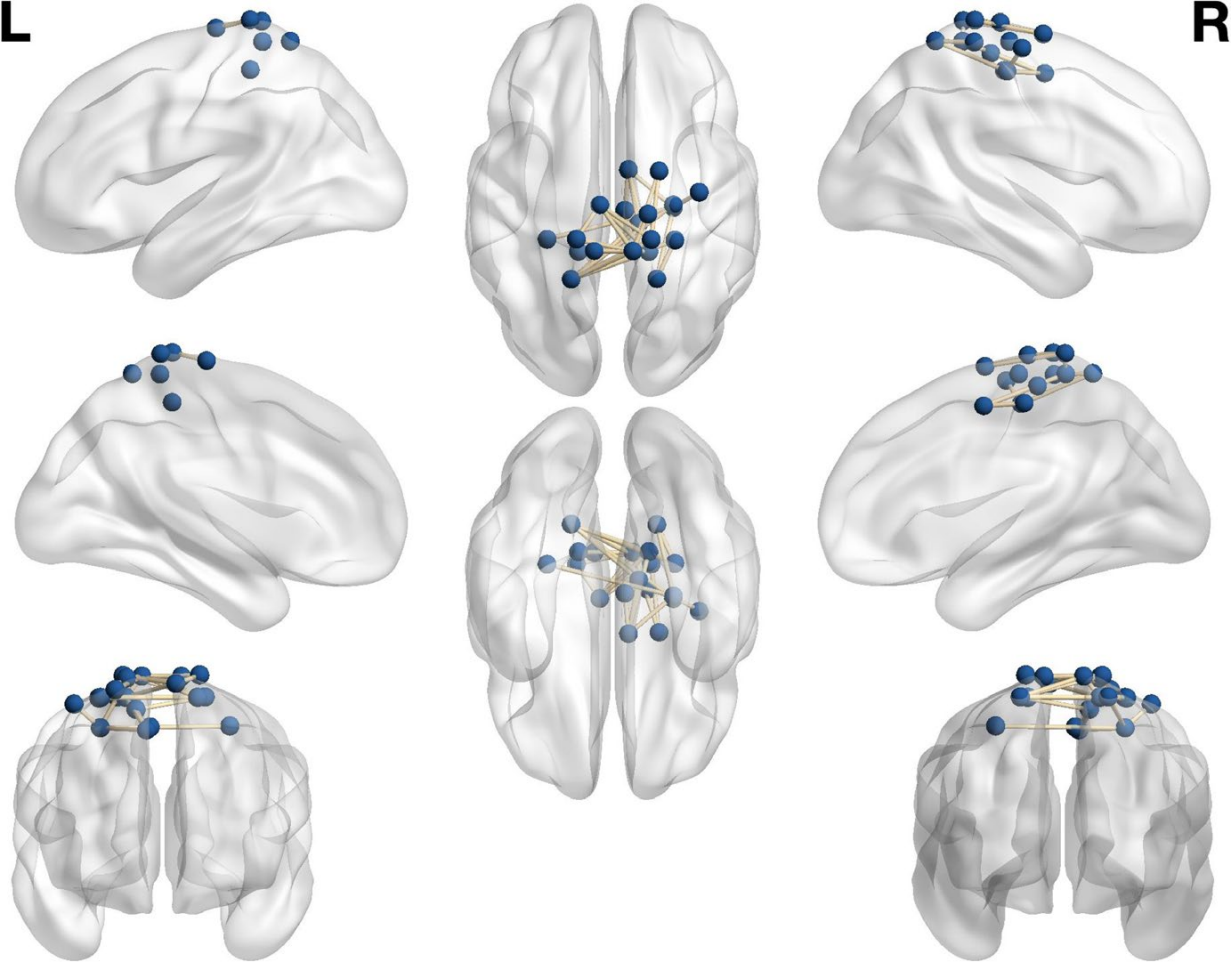
Significant network connections negatively related to “Self,” “Future,” and “Specific” thought content. Nodes visualized using BrainNet Viewer (Xia et al., 2013)

A positive correlation (*p* = .0081, .0486 FDR; Cohen's d = 0.58, initial threshold = 4.7) was observed between intrasubject network connectivity and partial factor scores for PC1 via the permutation‐based GLM. As the *factor loadings* were negative for this component, this finding suggests that network connectivity was negatively associated with thought content described by PC1: “self,” “future,” and “specific.” Brain regions therefore negatively correlated with PC1 include precentral gyrus, postcentral gyrus, superior parietal lobule, and superior frontal gyrus (Figure 3; see Tables 1 and 2 for MNI coordinates and full list of network connections). No significant relationships were observed between whole‐brain connectivity and PC2 or PC3.

**Table 1:** Node regions of Network 1

Atlas label	Hemisphere	MNI Coordinates		
		X	Y	Z
Postcentral gyrus 02	L	−14.58	−39.62	65.78
Postcentral gyrus 05	L	−7.87	−39.27	74.46
Precentral Gyrus 15	L	−5.52	−19.86	71.55
Superior Parietal Lobule 08	L	−27.92	−34.42	53.87
Superior Parietal Lobule 09	L	−15.42	−34.39	75.06
Superior Parietal Lobule 14	L	−17.66	−51.44	65.96
Juxtapositional Lobule Cortex (Supplementary Motor Cortex) 01	R	6.69	−4.86	52.19
Postcentral gyrus 02	R	15.39	−39.74	65.79
Postcentral gyrus 04	R	26.43	−34.79	65.5
Postcentral gyrus 05	R	8.6	−39.46	74.54
Postcentral gyrus 15	R	16.36	−34.54	74.8
Precentral Gyrus 02	R	25.84	−20.87	53.1
Precentral Gyrus 04	R	11.02	−27.78	60.74
Precentral Gyrus 06	R	15	−23.75	73.83
Precentral Gyrus 07	R	5.18	−22.09	53.4
Precentral Gyrus 12	R	25.82	−19.77	65.74
Precentral Gyrus 13	R	36.76	−14.45	62.96
Superior Frontal Gyrus 02	R	19.68	−5.47	68.98
Superior Parietal Lobule 04	R	18.76	−51.26	65.97

Note

Atlas regions and MNI Coordinates for all unique atlas regions contained within the significant network negatively related to PC1: “Self,” “Future,” and “Specific” thought content.

**Table 2:** List of network connections, Network 1

Direct Atlas Region Connections	
R Precentral Gyrus 02	R Precentral Gyrus 13
R Superior Frontal Gyrus 02	R Postcentral gyrus 05
R Precentral Gyrus 06	R Postcentral gyrus 05
R Postcentral gyrus 02	R Postcentral gyrus 05
R Superior Frontal Gyrus 02	R Postcentral gyrus 15
R Postcentral gyrus 05	R Postcentral gyrus 15
R Precentral Gyrus 02	R Superior Parietal Lobule 04
R Postcentral gyrus 04	R Superior Parietal Lobule 04
R Precentral Gyrus 02	R Juxtapositional Lobule Cortex (Supplementary Motor Cortex) 01
R Precentral Gyrus 04	R Juxtapositional Lobule Cortex (Supplementary Motor Cortex) 01
R Precentral Gyrus 07	R Juxtapositional Lobule Cortex (Supplementary Motor Cortex) 01
R Postcentral gyrus 02	R Juxtapositional Lobule Cortex (Supplementary Motor Cortex) 01
R Postcentral gyrus 02	L Precentral Gyrus 15
R Postcentral gyrus 05	L Precentral Gyrus 15
R Postcentral gyrus 15	L Precentral Gyrus 15
R Postcentral gyrus 05	L Postcentral gyrus 02
R Postcentral gyrus 05	L Postcentral gyrus 05
R Precentral Gyrus 02	L Superior Parietal Lobule 08
R Postcentral gyrus 05	L Superior Parietal Lobule 09
L Precentral Gyrus 15	L Superior Parietal Lobule 09
R Precentral Gyrus 04	L Superior Parietal Lobule 14
R Precentral Gyrus 12	L Superior Parietal Lobule 14
R Postcentral gyrus 02	L Superior Parietal Lobule 14
R Postcentral gyrus 04	L Superior Parietal Lobule 14
R Postcentral gyrus 05	L Superior Parietal Lobule 14
R Postcentral gyrus 15	L Superior Parietal Lobule 14

Note

Atlas regions listed here are bidirectional direct connections within Network 1.

## 4 DISCUSSION

Multi‐factor analysis provides a powerful method for analyzing the variance structure of repeated‐measures data. The creation of partial factor scores allows for repeated measures estimation of data‐driven components, and thus, can organically and reliably measures the thought content of individuals at rest independent of individual variation. The first *principal component* was most closely associated with “default‐mode” like thought content: specific thoughts about the self and future planning. As the first component, this dimension accounted for the most variance of the behavioral data. This finding is quite interesting, as rsfMRI’s most robust functional network is often associated with similar self‐referential thought content, although it is unlikely that this functional network is simply a mischaracterization of the most common topic of self‐generated thought, as evidence points to the DMN being an intrinsic quality of the brain and not just a function of fMRI (Kucyi et al., 2018), and mostly determined by anatomy (Teipel et al., 2010). While the current study was not able to demonstrate connectivity positively correlated with self‐referential thoughts, these “DMN‐like” thoughts were anticorrelated with connectivity in sensorimotor cortices and adjacent functional areas. A similar relationship has been demonstrated previously between self‐referential thoughts and the somatosensory cortices in an investigation of non‐DMN sites of self‐referential thought by Davey et al. (2016). Specifically, these authors demonstrated that the intra‐ and superior parietal cortex, right somatosensory cortex (as well as ventral posterior insular‐cortex, mid superior temporal cortex, posterior parahippocampal‐cortex, right posterolateral thalamus, and a small area of dorsal superior prefrontal cortex) showed greater activation for rest‐alone compared with the self‐referential condition. These results indicate these areas were active during rest, but were not involved in self‐referential processes, coinciding with the results of the current study. However, the self‐referential condition utilized Davey et al. (2016) was elicited via a prompt, while thought content was measured in the current study without provocation.

The results of the current study further demonstrate that whole‐brain FC networks are significantly related to the content of organic and unconstrained self‐generated thought. Research in patient populations has explored the dynamic of individual variation on resting FC, for example, the effects of state and trait rumination on resting FC in depression (Rosenbaum et al., 2017). While “state” rumination was measured in a manor analogous to mind‐wandering in this investigation (with a postscan questionnaire), “trait” rumination was determined using a clinical questionnaire. The results of the current study suggest that to adequately measure the effect state and trait rumination, subjects could simply repeat the resting‐state paradigm on different days and respond to an identical postscan questionnaire. Trait rumination, therefore, could be measured across these time points as opposed to estimation by a static clinical variable.

## 5 CONCLUSION

Continued research into both the stability and variability of FC networks and the functional representation of the content of mind‐wandering is necessary to untangle the influence of thought content, pathology, and FC. These issues are immediately relevant, as rsfMRI continues to be a standard paradigm in clinical research and is gaining popularity as a potential tool for clinical practice (O'Connor & Zeffiro, 2019).

## 6 STUDY LIMITATIONS

The intrasubject experimental design utilized in this study allows for control of known influences on BOLD and FC, such as age and sex. As well, this design eliminates possible confounds from task‐subject (thought‐subject) interactions outlined by Gratton et al. (2018). However, the current study was limited to 22 healthy individuals by the availability of the data present in this unique experimental design. Despite a large number of independent observations (88 total scans), the number of individuals included in this study is a notable limitation. The results of this study are further limited by the fact that these effects were only investigated in a single group of neurotypical adults. The intra‐subject variability of mind‐wandering and related FC may in fact differ across neuropsychiatric groups, and this is an effect which future research should quantify.

Functional neuroimaging is fundamentally confounded by a multitude of factors related to the measurement of BOLD such as: respiration, heart rate, and motion artifacts. These factors have been addressed to the best of our ability through canonical preprocessing steps such as motion correction and image (TR) censoring, regression of heart rate, and regression of respiration. While the necessity of these steps is well documented (Power et al., 2015, 2020), interpretation of BOLD fMRI and FC are further limited by the inclusion of preprocessing as the influence of these steps on neuroimaging results is not fully documented. We acknowledge the potential consequences of preprocessing choices in this research and suggest careful interpretation of the results presented here given these limitations.

## CONFLICT OF INTEREST

The authors declare no conflicts of interest.

## AUTHOR CONTRIBUTIONS

D.B., J.M, and L.M. contributed to the design and implementation of the research, to the analysis and interpretation of the results and to the writing of the manuscript.

## ETHICAL APPROVAL

The analyses presented in this study utilize publicly available, deidentified MRI images, and associated behavioral data; this study therefore does not constitute human subjects research.

## DISCLOSURES

In the past 5 years, Dr. Murrough has provided consultation services and/or served on advisory boards for Allergan, Boehreinger Ingelheim, Clexio Biosciences, Fortress Biotech, FSV7, Global Medical Education (GME), Impel Neuropharma, Janssen Research and Development, Medavante‐Prophase, Novartis, Otsuka, and Sage Therapeutics. In the past 12 months, Dr. Murrough has provided consultation services and/or served on advisory boards for Boehreinger Ingelheim, Clexio Biosciences, Global Medical Education (GME), and Otsuka. Dr. Murrough is named on a patent pending for neuropeptide Y as a treatment for mood and anxiety disorders and on a patent pending for the use of ezogabine and other KCNQ channel openers to treat depression and related conditions. The Icahn School of Medicine (employer of Dr. Murrough) is named on a patent and has entered into a licensing agreement and will receive payments related to the use of ketamine or esketamine for the treatment of depression. The Icahn School of Medicine is also named on a patent related to the use of ketamine for the treatment of PTSD. Dr. Murrough is not named on these patents and will not receive any payments.

### Peer Review

The peer review history for this article is available at https://publons.com/publon/10.1002/brb3.1860.

## Supporting information

Table S1

## Data Availability

All data, scan parameters, and descriptions of the experimental design have been reproduced from the data source: Gorgolewski et al. (2015). The data that support the findings of this study are openly available in the Consortium for Reliability and Reproducibility (CoRR) at http://dx.doi.org/10.15387/fcp_indi.corr.mpg1 (Gorgolewski & Margulies, 2015).
