# Supplementary material for: Intrasubject functional connectivity related to self‐generated thoughts
Source: Brain Behav. 2020 Dec 15;11(1):e01860. doi: 10.1002/brb3.1860 (PMC7821575; doi:10.1002/brb3.1860)
Supplement: Supplementary file 1 — Table S1 [file BRB3-11-e01860-s001.docx]

| **Supplementary Table 1.**  ***Mini-New York City Questionnaire*** | | |
| --- | --- | --- |
| NYC-Q Item | English Translation | In-Scanner Prompt (German) |
| positive | "I thought about something positive" | "habe ich an etwas Positives gedacht." |
| negative | "I thought about something negative" | "habe ich an etwas Negatives gedacht." |
| future | "my thoughts involved future events" | "habe ich an zukünftige Ereignisse gedacht." |
| past | "my thoughts involved past events" | "habe ich an vergangene Ereignisse gedacht." |
| myself | "my thoughts involved myself" | "habe ich über mich selbst nachgedacht." |
| people | "my thoughts involved other people" | "habe ich an andere Menschen gedacht." |
| surroundings | "my thoughts involved my surroundings" | "habe ich über meine derzeitige Umgebung nachgedacht." |
| vigilance | "I was fully awake" | "war ich vollkommen wach." |
| images | "my thoughts were in the form of images" | "hatte ich Gedanken in Form von Bildern." |
| words | "my thoughts were in the form of words" | "hatte ich Gedanken in Form von Worten." |
| specific_vague | "my thoughts were more specific than vague" | "waren meine Gedanken eher spezifisch als vage." |
| intrusive | "my thoughts were intrusive" | "waren meine Gedanken aufdringlich/eindringlich." |
